# Protective effect of *Glycyrrhiza glabra* roots extract on bone mineral density of ovariectomized rats

**DOI:** 10.1051/bmdcn/2019090208

**Published:** 2019-05-24

**Authors:** Dimitrios Galanis, Konstantinos Soultanis, Pavlos Lelovas, Alexandros Zervas, Panagiotis Papadopoulos, Antonis Galanos, Katerina Argyropoulou, Maria Makropoulou, Anastasia Patsaki, Christina Passali, Anastasia Tsingotjidou, Stavros Kourkoulis, Sofia Mitakou, Ismene Dontas

**Affiliations:** 1 Laboratory for Research of the Musculoskeletal System (LRMS), School of Medicine, National and Kapodistrian University of Athens, KAT Hospital Athens Greece; 2 1st Department of Orthopaedics, National and Kapodistrian University of Athens, Faculty of Medicine, Attiko Hospital Athens Greece; 3 Department of Pharmacognosy and Natural Products Chemistry, Faculty of Pharmacy, National and Kapodistrian University of Athens Greece; 4 Hellenic Red Cross Hospital Athens Greece; 5 Lab. of Anatomy, Histology and Embryology, School of Veterinary Medicine, Faculty of Health Sciences, Aristotle University of Thessaloniki School of Veterinary Medicine; 6 Department of Mechanics, National Technical University of Athens (NTUA), National Technical University of Athens Greece

**Keywords:** Rat, Osteoporosis, Ovariectomy, Absorptiometry, Three-point-bending

## Abstract

Objective: The aim of this study was to evaluate the potential effect of the methanolic extract of plant *Glycyrrhiza glabra* roots on bone mineral density and femoral bone strength of ovariectomized rats.

Methods: Thirty 10-month-old Wistar rats were randomly separated into three groups of ten, Control, Ovariectomy and Ovariectomy-plus-Glycyrrhiza in their drinking water. Total and proximal tibial bone mineral density was measured in all groups before ovariectomy (baseline) and after 3 and 6 months post ovariectomy. Three-point-bending of the femurs and uterine weight and histology were examined at the end of the study.

Results: No significant difference was noted in bone density percentage change of total tibia from baseline to 3 months between Control and Ovariectomy-plus-Glycyrrhiza groups (+5.31% ± 4.75 and +3.30% ± 6.31 respectively, *P* = non significant), and of proximal tibia accordingly (+5.58% ± 6.92 and +2.61% ± 13.62, *P* = non significant) demonstrating a strong osteoprotective effect. There was notable difference in percentage change of total tibia from baseline to 6 months between groups Ovariectomy and Ovariectomy-plus-Glycyrrhiza (−13.03% ± 5.11 and −0.84% ± 7.63 respectively, *P* < 0.005), and of proximal tibia accordingly (−27.9% ± 3.69 and −0.81% ± 14.85 respectively, *P* < 0.001), confirming the protective effect of *Glycyrrhiza glabra* extract in preserving bone density of the Ovariectomy-plus-Glycyrrhiza group. Three-point-bending did not reveal any statistically significant difference between Ovariectomy and Ovariectomy-plus-Glycyrrhiza groups. Uterine weights of the Ovariectomy-plus-Glycyrrhiza group ranged between the other two groups with no statistically significant difference to each.

Conclusions: *Glycyrrhiza glabra* root extract notably protected tibial bone mineral density loss in Ovariectomy-plus-Glycyrrhiza rats in comparison with ovariectomized rats, but did not improve biomechanical strength.

## 1. Introduction

Postmenopausal osteoporosis is a known health problem that affects mostly white women worldwide. Disturbance of trabecular bone architecture and cortical weakening resulting in significant loss of bone mechanical strength increases the incidence of lumbar, forearm and hip fractures, the latter being a serious socioeconomic issue since operative treatment is almost always mandatory and mortality rate at one year exceeds 15-30% [1–6].

The necessity of pharmacological osteoprotective intervention (hormone therapy, calcitonin, biphosphonates, strontium ranelate, Selective Estrogen Receptor Modulators) is widely considered as the optimal mode to confront osteoporosis [7–9]. Long term compliance however can be low [10–13] due to of biological adverse effects and complications, such as coronary heart disease, stroke, breast and uterus cancer, low energy fractures of the femur and osteonecrosis of the jaw [14–19]. The desire to cope with osteoporosis using non- pharmaceutical protective regimen becomes even stronger as the indications of the beneficial effect of certain plants lead to studies of plants extracts, some of which display phytoestrogenic properties [20–23]. Phytoestrogens are plant derived phenolic compounds exerting osteoprotective properties [21–24]. Epidemiologic data also show a lower incidence in breast and prostate cancer in phytoestrogen consuming populations [24]. Furthermore *in vitro* and animal studies set up an important anteroom for successful clinical trials, which desirably would lead to complication free management of osteoporosis [25–30].

*Glycyrrhiza glabra* (G.glabra), a plant also referred to as liquorice with a traceable history of 6000 years [31] is a herbaceous perennial plant indigenous to southern Europe, India and parts of Asia, reaching 1.2 m by 1 m and has been widely utilized for its roots, which reveal a sweet flavor, on account of multi-active substance glycyrrhizin [32, 33]. Previous research on *Glycyrrhiza glabra* also confirmed the presence of phytooestrogens and other constituents isolated from liquorice roots with oestrogenic-like activity [34]. *Glycyrrhiza glabra* properties are not restricted to bone protection but also display a variety of desirable biological effects such as anti-lipidaemic [35–40], hypo-cholesterinaemic [41] and anti-diabetic [42] actions. Although liquorice abuse can be harmful, its low toxicity in normal consumption render *Glycyrrhiza glabra* a healthy food source [32]. Furthermore liquorice is being largely utilized as a sweetener during food and beverage preparation and as important ingredient in cosmetics, pharmacology and tobacco industry [43–46].

The fact that *in vitro* and *in vivo* studies documented the varying levels of estrogen receptor (ER) agonism of liquorice root extract in different tissues [34], led us to further investigate the estrogen-like activity on bone *in vivo*. Our aim was to assess the potential beneficial action of *Glycyrrhiza glabra* extract (G) on bone of adult rats subjected to experimentally-induced osteoporosis, which is the commonly used animal model for the study of postmenopausal osteoporosis [47]. The possible effects on the uterus were also analyzed in the study.

## 2. Materials and methods

### 2.1. Laboratory animals

The General Directorate of Veterinary Services approved our experimental protocol (permit no. K4505/10-7-2014), according to national legislation (Presidential Decree 56/2013, in conformance with the European Directive 2010/63/EU).

The registered breeding unit of the Hellenic Pasteur Institute (Athens, Greece) provided us with 30 10-month-old intact mature female Wistar rats. The Wistar rat is an outbred stock. The animals were placed three or four in a cage (dimensions 45 × 30 × 20 cm; IFFA), in the controlled enviromental conditions of the animal house, with temperature 19-22° Celsius, relative humidity 55% to 65%, fifteen air changes per hour, and a light/dark cycle of 06:00/18:00 hours. The rats underwent a baseline body weight measurement and afterwards were allocated randomly into three groups, Control (n = 10), Ovariectomy (OVX, n = 10), OVX- plus-Glycyrrhiza (OVX + G, n = 10). Body weight and littermates were taken into account in groupings to minimize possible genetic variations. The body weights and the food consumption were measured at least once per week.

### 2.2. Measurements of Bone Mineral Density

A General Electric Lunar Prodigy Densitometer was used for the assessment of bone mineral density (BMD) by Dual-energy X-ray absorptiometry (DXA), and making use of a dedicated small animal software. Initial system calibration was performed before every group measurement. Anesthesia before each measurement took place with use of dexmedetomidine and ketamine. All rats were measured initially before any intervention and at three and six months post-OVX. The regions of interest (ROIs) defined for the proximal tibia measurements were squares sized 0.16 × 0.16 cm. One blinded observer recorded the values.

### 2.3. Ovariectomy

The Sham-operated control group was used in order to evaluate the BMD of age matched non-OVX rats to allow comparison in relation to the BMD changes of OVX rats with and without therapy. Ovariectomy in groups OVX and OVX + G was performed after the initial BMD measurement. Anesthesia and analgesia was applied by intramuscular injection of dexmedetomidine / ketamine and carprofen respectively, bilateral OVX was carried out with the midline approach under aseptic procedures. The peritoneum and skin incisions were closed separately with single interrupted sutures.

### 2.4. Extract

### 2.5. Analysis

Ultra Performance Liquid Chromatography - High Resolution Mass Spectrometry (UPLC-HRMS) & High Resolution Mass Spectrometry / Mass Spectrometry (HRMS/MS) analysis of *Glycyrrhiza glabra* An AQUITY UPLC system (Waters), which was connected to an LTQ-OrbitrapR XL hybrid mass spectrometer (Thermo Scientific), was used to perform the LC-MS analysis. Electrospray ionization (ESI) was used and the operation was performed in negative mode.

The solvent system consisted of (A) water solution of 0.1% formic acid and (B) acetonitrile, and the flow rate was set at 0.4 *ml*/min. The following program followed for elution: 2% B for 2 minutes; 100% B for 18 minutes; stop for 2 minutes; 2% B for 1 minute. Column equilibration was carried out at the end of the run for 4 minutes. Moreover, injection volume was set to 10 μL. Water/acetonitrile (1 : 1) was used for sample preparation at a concentration of 0.3 mg/*ml*. Supelco Ascentis Express C18 (100 x 2.1 mm i.d, 2.7 μm particle size) chromatographic column was used. All data gained from HRMS & HRMS/MS was acquired over a mass range of 100–1000 m/z. The following ESI conditions were applied: capillary temperature 320 °C; capillary voltage −40 V; tube lens −120 V; ESI voltage 2.7 kV.

Nitrogen served both as sheath and auxiliary gas (40 Au and 8 Au, respectively). For the HRMS/MS acquisitions, a data- dependent method was used, comprising the detection (full scan) and fragmentation of the three most intense peaks for each scan. Regarding the HRMS/MS experiments, the mass resolving power was 30.000 for all levels and the regularized collision energy in the ion trap was set to 35.0% (q = 0.25). In order to identify the extract constituents (*e.g* retention time, accurate m/z, polarity, proposed elemental composition, ring double bond equivalent values and HRMS/MS spectra and derived fragmentation motifs) chromatographic and spectrometric characteristics were used. The raw data were obtained and processed with use of the XCalibur software version 2.2.4 by Thermo Scientific.

High Performance Liquid Chromatography - Photodiode Array (HPLC-PDA) analysis of *Glycyrrhiza glabra*.

For the HPLC analysis of the methanol extract of liquorice, a Thermo Finnigan^®^ HPLC-PDA System (P4000 Pump, AS3000 Autosampler, PDA Detector UV8000, ChromquestTM 4.1 Software) and a Supelco^®^ RP18 Discovery HS-C18 (250 mm, 4.6 mm, 5 μm) chromatographic column were utilized. The mobile phase was 0.1% formic acid (Solvent A) and methanol (Solvent B). The elution was initiated with 2% (B), reached 40% (B) in 10 min and held isocraticaly for 10 min; 50% (B) in the next 5 min and held isocraticaly for 10 min; 100% (B) in 25 min and held for 5 min before returning to initial conditions in 2 min, for a 5- minute re-equilibration. The detection was performed at 254 nm, 280 nm and 366 nm. The flow rate was kept stable at 1 *ml*/min and the column temperature at 25 °C.

For identification of the active compounds in *G. glabra* extract, a LC-HRMS method was applied. Specifically, an Orbitrap mass analyzer of high resolution and in negative ionization mode was used. Identification of peaks was performed by comparing the HRMS and HRMS/MS data of the detected constituents with relevant published data [48–50] and natural products databases (PubChem, ChemSpider).

**Fig. 1:**
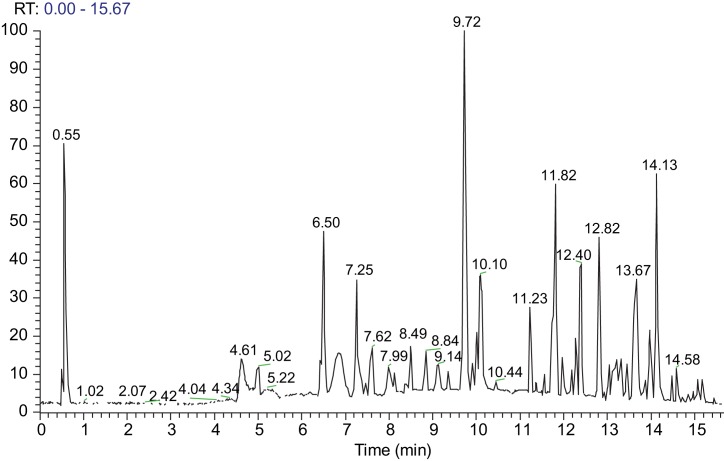
UPLC-ESI(-)-HRMS full scan chromatogram of the methanolic extract of *G. glabra*. The respective compounds are listed in Table 1. RT = Retention Time.

Additionally, the relative quantitative analysis of the extract was carried out using a method developed on an HPLC-DAD system. The extract was particularly rich in phenolic constituents and saponins. Glycyrrhizin was the major constituent of liquorice, with a concentration of 12.8% in the methanol extract. Liquiritin apioside (4.1%), 3-hydroxyglabrol (3.2%), glabridin (3.1%) and kanzonol Y (3.0%) were the other main compounds in the extract. The chromatogram of the methanolic extract of *G. glabra* is demonstrated in Fig. 1. The compounds detected and identified in the methanol extract of *G. glabra* using UPLC-ESI-HRMS & HRMS/MS is listed in Table 1.

**Table 1:** Chromatographic and spectrometric characteristics of compounds identified in the methanol extract of *G. glabra* using UPLC-ESI(-)-HRMS.

[M-H]-						
Compounds	t_R_ (min)	Theoretical m/z	Experimental m/z	Delta (ppm)	RDBeq	Molecular Formula
Liquiritin apioside	6.50	549.16136	549.16168	0.58	12.5	C_26_H_30_O_13_
Licorice-glycoside C1						
or	7.62	725.20825	725.20871	−0.63	18.5	C_36_H_38_O_16_
Licorice-glycoside C2						
Naringenin	7.99	271.06120	271.06140	0.75	10.5	C_15_H_12_O_5_
Licorice glycoside A	8.13	725.20871	725.20825	−0.63	18.5	C_36_H_38_O_16_
Echinatin	8.39	269.08193	269.08215	0.82	10.5	C_16_H_14_O_4_
Licorice saponin G2	8.84	837.39142	837.39099	−0.52	12.5	C_42_H_62_O_17_
Liquiritigenin	9.14	255.06628	255.06654	1.03	10.5	C_15_H_12_O_4_
Formononetin	9.36	267.06628	267.06650	0.81	11.5	C_16_H_12_O_4_
Glycyrrhizic acid	9.72	821.39651	821.39612	−0.48	12.5	C_42_H_62_O_16_
Glycybridin J	9.91	369.13436	369.13446	0.27	11.5	C_21_H_22_O_6_
Licorice saponin B2	10.10	807.41724	807.41748	0.29	11.5	C_42_H_64_O_15_
Licorice saponin K2/H2	10.13	821.39541	821.39636	1.16	12.5	C_42_H_62_O_16_
Licoflavone C	10.44	337.10815	337.10831	0.47	12.5	C_20_H_18_O_5_
Erybacin B	11.23	325.10815	325.10834	0.59	11.5	C_19_H_18_O_5_
Glabrone	11.37	335.09250	335.09259	0.28	13.5	C_20_H_16_O_5_
Kanzonol A	11.56	339.12380	339.12375	−0.14	11.5	C_20_H_20_O_5_
Kanzonol W	11.72	335.09250	335.09262	0.37	13.5	C_20_H_16_O_5_
Glabridin	11.82	323.12888	323.12906	0.55	11.5	C_20_H_20_O_4_
Glabrene	11.97	321.11323	321.11337	0.44	12.5	C_20_H_18_O_4_
Kanzonol Y	12.40	409.20205	409.20209	0.10	11.5	C_25_H_30_O_5_
Glycybridin C	12.82	409.20205	409.20227	0.55	11.5	C_25_H_30_O_5_
Phaseollin	13.67	643.23374	643.23376	0.04	23.5	C_40_H_36_O_8_
3-Hydroxyglabrol	14.13	407.18640	407.18671	0.76	12.5	C_25_H_28_O_5_
Glabrol	14.58	391.19148	391.19153	0.12	12.5	C_25_H_28_O_4_

### 2.6. Preparation

*Glycyrrhiza glabra* dried roots were extracted with Methanol (MeOH) using Ultrasound assisted extraction method and the dried extract was dissolved in drinking water. The concentration of this water was 2 mg/*ml*.

### 2.7. Administration

*Glycyrrhiza glabra* was consumed by the OVX + G group in their drinking water one day post OVX. The extract volume to be consumed was recalculated anew every 3 days according to the monitoring of fluid consumption per cage adjusted per rat. The approximate desired concentration consumed per rat was calculated by taking into account the minimum and maximum proposed consumption of comminuted herbal substance of an average human per day, using the European Medicines Agency instructions, which is set to 3000-8000 mg per day for humans contained in 500-1000 *ml* per day [51]. In addition and by further focusing on Glycyrrhizin, the main active substance of *G. glabra*, its maximum daily adverse-effect-free dosage based on an older study with female volunteers was reported as 2 mg/kg/day [32], which is still used as reference in recent publications. The dosage in our study was constantly recalculated throughout the study based on the body weight changes of the rats, using a published algorithm for conversion of human dosage to animal dosage [52], which in our case was converted to a desired upper threshold of 12.4 mg/ kg of Glycyrrhizin per rat per day.

Our goal was to stay under the maximum allowed limits of Glycyrrhizin consumption according to European Medicines Agency instructions. Glycyrrhizin demonstrates poor bioavailability after oral intake [32] and it can be found at 10-20% in Licorice fluid extracts [32]. Based on the above and redefining the extract concentration every three days, the consumption of the rats in our study did not exceed the upper limits of Licorice consumption. Additionally we did not observe any clinical adverse effect in the OVX + G rats during the study.

### 2.8. Collection of tissues

Euthanasia was performed with use of anesthesia overdose and exsanguination from the abdominal aorta. All animals underwent autopsy in order to check the success of OVX and to further assess the tissues for possible abnormal pathological findings, *e.g.* malignancies, hypertrophy *etc*. The criteria used to confirm successful OVX were detection of uterine horn atrophy and absence of visible traces of ovarian remnants. The uteri were dissected free of the adjacent tissues and weighed immediately after autopsy. Both femurs were removed to undergo further mechanical strength testing.

### 2.9. Biomechanical testing

Mechanical bone strength was assessed *ex vivo* after euthanasia using the three-point- bending (3 PB) method. Both femurs of each rat were collected free of other tissues, then wrapped in gauzes soaked in normal saline and preserved at −20 °C until the day of testing seven days later. This is the indicated method for long-term preservation before biomechanical testing [53]. An MTS 858 Mini Bionix frame was utilized for the tests. The equipment was initialized and calibrated at the beginning of each testing day. A blind observer performed the tests and the output values were automatically archived in the computer to be collected and analyzed.

Each femur was placed on two blunt edges which had a distance of 22 mm. All femurs were placed in the exact same manner in terms of orientation and rotation stability. Specimens which rotated during loading were not included in the data analysis. Vertical load was applied through a punching rounded notch at the midshaft of the diaphysis continuously until fracture. Displacement rate was set to 1 mm / minute. The maximal load at fracture was recorded and a graph regarding the relation between load and displacement was exported.

### 2.10 Statistical analysis

Data were presented as mean ± standard deviation (SD). The Shapiro-Wilks test was used for the normality analysis of the parameters.

The comparison of ΒΜD parameters at each time point was done using the One way ANOVA model. The Bonferroni test was used for the pairwise comparisons.

One factor Repeated Measures ANOVA model was utilized for the comparison of different time measurements of BMD parameters for each group. The Bonferroni test was used for the pairwise multiple comparisons.

To indicate the trend in 6 months of observation/treatment, the mean percentage changes from baseline to 3 and 6 months respectively were calculated. Comparison of percentage change from baseline of BMD parameters during the observation period between the groups was analyzed using the One way ANOVA model. The Bonferroni test was used for the pairwise multiple comparisons. In cases where there was normality violation the tests Kruskal Wallis and Mann-Whitney were utilized.

Two-way Analysis of variance model was used for the analysis of Maximum Load variable using as factors the “treatment group” (Control – OVX – OVX + G) and the “Leg” (right - left). The results were presented as mean ± standard error (SE).

All tests are two-sided. The statistical significance was defined at *P* < 0.05. For all the data analyses the statistical package of SPSS version 17.00 was utilized (Statistical Package for the Social Sciences, SPSS Inc., Chicago, Ill., USA).

## 3. Results

### 3.1. BMD absolute values

#### 3.1.1. Total tibia

**Fig. 2:**
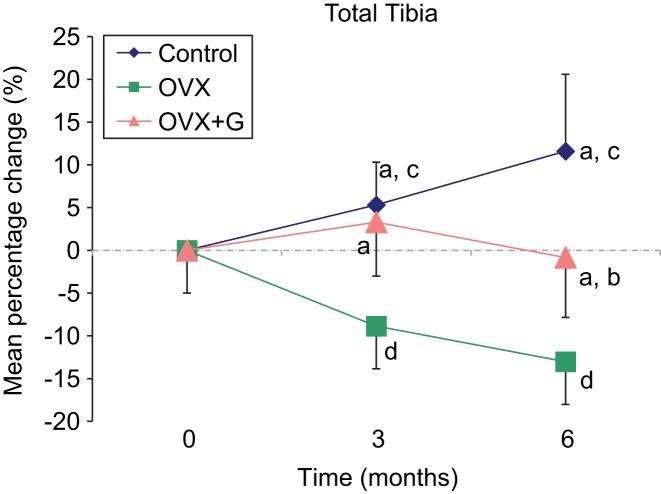
Graphical presentation of the mean percentage changes of total tibia BMD from baseline to 3 and 6 months of the three groups. a: *P* < 0.005 *vs*. OVX, b: *P* = 0.005 *vs*. Control, c: *P* < 0.05 *vs.* baseline, d: *P* < 0.001 *vs.* baseline. Control n = 10, OVX n = 8, OVX + G n = 8. OVX: Ovariectomy; G: *Glycyrrhiza glabra* extract.

The BMD measurements of the total tibia, carried out pre-OVX, three and six months post-OVX by DXA for the three groups, are shown in Table 2 and Fig. 2 At 3 months the OVX group shows a significant decrease of BMD. On the contrary the, OVX + G group has similar values to the Control group, maintaining the BMD of the total tibia. At 6 months the Control group presents a further BMD increase and the OVX group a further BDM decrease, both significantly different compared to their respective baseline values. The OVX + G group decreases its BMD as well but less than the OVX animals which indicates a protective effect from the plant extract.

**Table 2:** Comparison of absolutes values (g/cm^2^) and mean percentage changes from baseline of the total tibia BMD among the three groups measured at baseline (before OVX), three and six months after OVX. BMD: bone mineral density; OVX: ovariectomy; G: *Glycyrrhiza glabra* extract. SD: Standard Deviation.

Total tibial BMD								
Group	Baseline, mean ± sd	3 months, mean ± sd	6 months, mean ± sd	*P* value within group	% change baseline-3 months, mean ± sd	*P* value between groups 3 months	% change baseline-6 months, mean ± sd	*P* value between groups 6 months
Control	0.214 ± 0.032	0.215 ± 0.010	0.228 ± 0.015	0.224	5.31 ± 4.75		11.59 ± 9.12	
OVX	0.225 ± 0.015	0.204 ± 0.007	0.195 ± 0.009	0.040	−8.87 ± 3.80	= 0.003	−13.03 ± 5.11	< 0.001
OVX + G	0.209 ± 0.013	0.215 ± 0.016	0.206 ± 0.017	<0.0005	3.30 ± 6.31		−0.84 ± 7.63	

#### 3.1.2. Proximal tibia

**Fig. 3:**
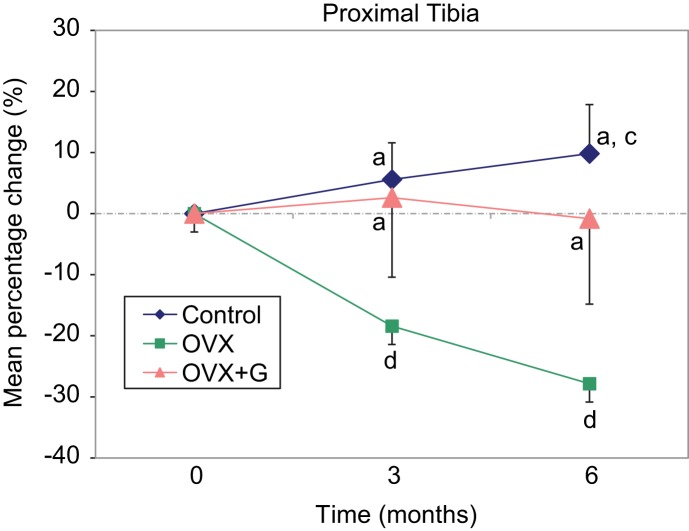
Graphical presentation of the mean percentage changes of proximal tibia BMD from baseline to 3 and 6 months of the three groups a: *P* < 0.001 *vs.* OVX, c: *P* < 0.05 *vs.* baseline, d: *P* < 0.001 *vs.* baseline. Control n = 10, OVX n = 8, OVX + G n = 8. OVX: Ovariectomy; G: *Glycyrrhiza glabra* extract.

The BMD measurements of the proximal tibia are presented in Table 3 and Fig. 3. The osteoprotective effect of *Glycyrrhiza glabra* is also observed at this bone site, since the BMD of the OVX + G group remains similar to the Controls’ at 3 months and close to baseline at 6 months (0.427 ± 0.025 *vs*. 0.390 ± 0.082 and 0.410 ± 0.019 *vs*. 0.406 ± 0.073 respectively, *P* = non significant), whereas the BMD of the OVX group declines significantly (0.332 ± 0.019 at 3 months and 0.293 ± 0.011 at 6 months, *P* < 0.001 between baseline, three and six months).

**Table 3:** Comparison of absolutes values (g/cm^2^) and mean percentage changes from baseline of the proximal tibia BMD among the three groups measured at baseline (before OVX), three and six months after OVX. BMD: bone mineral density; OVX: ovariectomy; G: *Glycyrrhiza glabra* extract. SD: Standard Deviation.

Proximal tibial BMD								
Group	Baseline, mean ± sd	3 months, mean ± sd	6 months, mean ± sd	*P* value within group	% change baseline-3 months, mean ± sd	*P* value between groups 3 months	% change baseline- 6 months, mean ± sd	*P* value between groups 6 months
Control	0.390 ± 0.025	0.410 ± 0.019	0.427 ± 0.025	0.002	5.58 ± 6.92		9.84 ± 8.63	
OVX	0.407 ± 0.020	0.332 ± 0.019	0.293 ± 0.011	<0.001	−18.42 ± 3.05	< 0.001	−27.86 ± 3.69	< 0.001
OVX + G	0.394 ± 0.035	0.406 ± 0.073	0.390 ± 0.082	<0.663	2.61 ± 13.62		−0.81 ± 14.85	

### 3.2. BMD percentage changes

No considerable difference was noted in BMD percentage change of the total tibia from baseline to 3 months between Control and OVX + G groups (+5.31% ± 4.75 and +3.30% ± 6.31 respectively, P = non significant) and of proximal tibia (+5.58% ± 6.92 and +2.61% ± 13.62, P = non significant) indicating a bone protective effect (Fig. 2 and Fig. 3 respectively). There was a notable difference in the BMD percentage changes between OVX and OVX + G groups of the total tibia, from baseline to 6 months (−13.03% ± 5.11 and −0.84% ± 7.63 respectively, *P* < 0.005) and of the proximal tibia (−27.86% ± 3.69 and −0.81% ± 14.85 respectively, *P* < 0.001) (Fig. 3), indicating a protective effect of *Glycyrrhiza glabra* extract in preserving the BMD of the OVX + G group.

### 3.3. Biomechanical tests

**Fig. 4:**
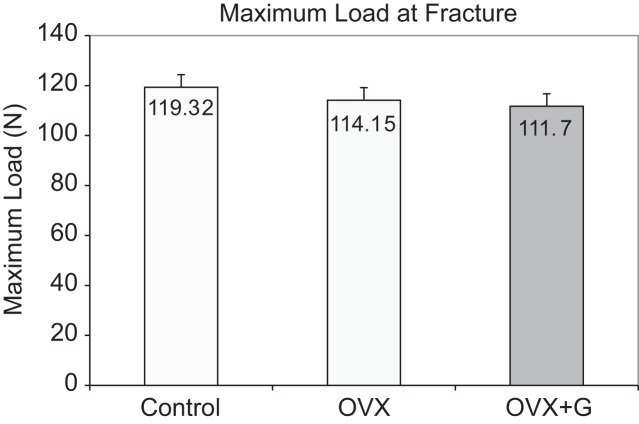
Mean values of maximum load at fracture (force measured in Newtons) of the femur in the three groups at the end of the study *ex vivo*. Respective Standard Error (SE): Control = 5.13, OVX = 4.83, OVX + G = 5.13. OVX: Ovariectomy; G: *Glycyrrhiza glabra* extract.

The results of the maximum force at fracture (in Newtons) for all the three groups is shown in Fig. 4. There was no statistically significant interaction between the factors group and right or left femur (*P* = 0.489).

Three-point bending did not reveal differences that were statistically significant between the three groups (*P* = 0.569)

### 3.4. Body weights

All three rat groups increased their body weight in parallel during the study with minor weight fluctuations. At baseline the rats had similar body weight and at the completion of the study their body weight values in order of magnitude were OVX > OVX + G > Control, without statistically significant differences. Two rats from the Control group and two from the OVX + G group did not recover from anesthesia during the BMD measurement at zero and three months respectively.

### 3.5. Uterine weight

The mean uterine weights of the three groups is shown in Table 4. The mean uterine weight of the Control group was statistically significantly higher than the OVX mean value (*P* = 0.015).

**Table 4:** Uterine weight (in grams) of the three groups. OVX: ovariectomy; G: *Glycyrrhiza glabra* extract. *P = 0.015 *vs.* Control. SD: Standard deviation

Uterine weight, grams				
Group	n	Mean	SD	*P* value between groups
Control	8	0.628	0.118	
OVX	10	0.350*	0.218	=0.015
OVX+G	8	0.526	0.205	

The mean uterine weight of the OVX + G group was between the other two groups with no statistically significant difference, neither with the Control group (*P* = 0.878), nor with the OVX group (*P* = 0.184).

### 3.6. Uterine histology

The uterine specimens were maintained in formalin. They were processed for paraffin embedding. Specimens were cut at 7 μm thickness at transverse and/or horizontal and sagittal sections. All different layers were present at the transverse sections. At some instances not all layers were included at the horizontal sections. H & E staining was performed at all cases. The three major divisions were distinguished (endometrium, myometrium and perimetrium) and evaluated.

Although the histology between the three groups was overlapping, there was a tendency in the treated uteri where the endometrium was thin with simple cuboidal epithelium and the subepithelium was densely packed with moderate glandular tissue of simple cuboidal epithelium. The rest of the layers were distinguishable and richly vascularized. The overall comparison between all groups showed that the glands were numerous in the Control rats, distinctive in the OVX group and limited in the OVX + G group. Additionally the uteri of the OVX group had glands of larger diameter, of which were not evident in the Control rats and much less existent in the OVX + G group. Further evaluation of the uteri of all three groups did not reveal any obvious sign of hypertrophy, inflammation or cellular alteration, thus indicating the absence of uterus-focused effects of the *G. glabra* extract.

## 4. Discussion

The present study investigated the effect of *Glycyrrhiza glabra* administration on ovariectomized rats. As to be expected the OVX group showed a significant decrease of BMD during the first 3 months which continued until 6 months. On the contrary the OVX + G group had similar BMD values to the Control group after 3 months of administration which demonstrated an increase of the BMD of both the total and proximal tibia, which at 6 months decreased to approximately baseline values. This tendency of the OVX + G group to maintain BMD levels indicates an osteoprotective effect of the *G. glabra* extract.

Similar studies demonstrating a bone-protective effect on the ovariectomized rat model by plant extracts have been published. The administration of Onobrychis plant extract showed statistically significant increased BMD values in the treated rats compared to the OVX rats (−12.75% *vs*. −27.65% respectively) [54]. Likewise, a 12 week high dose administration of *Drynariae rhizoma* extract in rats significantly increased the BMD in the treated animals in comparison to the OVX animals [55]. In addition, after three months of consumption of the citrus flavonoid Rutin, treated rats showed a significant increase in their BMD in comparison to the OVX group [56]. The plant extract from *Sideritis euboea* significantly preserved the proximal tibial BMD in treated rats compared to the OVX group, both from baseline to three and six months; there was a statistically significant difference in BMD percentage loss from baseline to three months and to six months between the OVX group and the treated group (−26.47% *vs*. −15.57% and −31.22% *vs*. −16.57% respectively) [57].

The three-point-bending test of our study did not reveal any statistically significant difference between the OVX and OVX + G groups probably due to the test being conducted in the femoral shaft which is cortical bone-dominated and slow in showing treatment effect. The average force applied until fracture was slightly lower in the OVX + G group than the OVX group which was unexpected. The femoral diaphysis consists mainly of cortical bone and in our animal model trabecular bone loss is what mainly occurs [58, 59], thus leaving the femoral diaphysis bone strength unaffected [58].

However, a 13 week study of tea polysaccharide administration to OVX rats demonstrated an improvement of femoral biomechanical properties to a certain extent [60]. Likewise the administration of the plant extract of *Sideritis euboea* in a similar to our 6 months study showed that the maximum load before fracture was statistically significantly higher in the treated group in comparison to the OVX group [57]. Similarly, the administration of *Dendrobium officinale* orchid extract in rats for 13 weeks showed that femoral maximum load was enhanced in the treatment group compared to the OVX group, although not significantly [60]. On the contrary, a study on ovariectomized rats about a phytoestrogen Diarylheptanoid concluded that this active substance protected the loss of only trabecular bone and not cortical [59], which is in agreement with our test results regarding femoral diaphysis mechanical bone strength using *G. glabra*. In another study of 5 months duration on an OVX model with 17alpha-ethinylestradiol, the authors examined the biomechanical quality of the femur and found that 17alpha-ethinylestradiol administration had no significant effect on measurements of energy and maximum load compared to the OVX group [61]. Furthermore a research study examining the effect of 12 week post OVX administration of flavonoids from *Drynariae rhizoma* in rats showed that although the maximum load force was higher in the Sham group, there was no significant effect on maximum load between treated group and OVX group [55]. It has also been reported that a decrease in bone strength of the femoral midshaft can be seen at the earliest at 9 months post OVX [62], which is later than the time of our testing.

In our study, OVX uterine weights were significantly lower in comparison to the Control group. The OVX + G group showed an increase in uterine weights in comparison to the OVX group.

They were however lower than the Controls’ weight and did not display any uterotrophic effect, which would be an undesirable action. In two 6 month studies similar to ours it was reported that the OVX group mean uterine weights were significantly lower in comparison to the Control group and identical to the treated group [54, 57]. The 13-week study regarding *Dendrobium officinale* also observed the same significant uterine weight loss in the OVX group, but the treated group showed significant enhanced uterine weights [60]. In another study, the 16-week administration of Du-Zhong cortex extract showed that the OVX group showed significant uterine weight decrease; in the treated animals, uterine weight was not different and additionally no uterotrophic effect was demonstrated [61].

Although the actions of the various components of the methanolic extract of *G. glabra* have been widely studied in many organs and tissues, there is little information directly related to bone properties. Nevertheless, there is some quite interesting information regarding the actions of some of these components on bones, all of which are listed in Table 1. Specifically, in a 2-month study the oral administration of Glycyrrhizic acid in 3-month-old rats protected the femoral bone against glucocorticoid-induced osteoporosis and led to improvement of the bone structure and resistance [63]. Another study using rats showed that Glabridin and Glycyrrhizic acid had a slightly positive effect on the osteoporotic bone tissue, while Glycyrrhizic acid had very little effect on the skeletal system [64]. It has been also documented that Glabridin has positive action on estrogen receptors, being a potential Selective Estrogen Receptor Modulator [65]. Furthermore Glabridin and Glabrene from licorice roots showed estrogenic activity on rat bone tissues in the prepubertal phase and on human osteoblasts contained in cell cultures from pre- and post- menopausal women [66]. In a recent *in vitro* study, Glycyrrhizin was found to have a suppressive action on RANKL- induced osteoclastogenesis, thus being a potentially effective antiosteoporotic regimen, as proposed by the authors of this study [67]. Another component of *G. glabra* root extract, the methoxy isoflavone named Formononetin, was found to promote the healing of the experimentally induced midfemoral bone injury and to significantly reinforce bone regeneration in female mice [68]. Interestingly, in a 12-week study, Formononetin showed the ability to inverse osteopenia and promote new bone formation in ovariectomized adult rats [69]. In a 4-week study where Wistar rats were used, it was documented that the flavonoid Naringenin had a small positive effect regarding bone microarchitecture in the osteoporotic tissues, but did not affect the mechanical and chemical properties of the bones [70]. Furthermore the flavonoid Liquiritigenin expressed a dual effect on bone cells by both promoting the differentiation of osteoblast and inhibiting the differentiation of osteoclasts [71]. Beside the actions on bone metabolism, most of the components of *G. glabra* extract show additional beneficial effects, some of which deserve a short reference. In the literature it has been described that the flavonoids acquired from Glycyrrhiza express anti-inflammatory, antioxidant and anti-bacteria action [72]. In addition Glycyrrhizic acid was found to protect the myocardium against ischemia [73] and to show strong neuroprotection in rodent model of chronic cerebral hypoperfusion [74]. Licoflavone protected the gastric mucosa in rats by up- regulating the serum levels prostaglandin E2 [75] and was also found to have anti-ulcer effect in rats through regulation of the inflammation mediators and the metabolism of amino acid [76]. Furthermore Glabrone demonstrated antiviral activity [77], while Glabrol showed a potential antihypercholesterolemic effect [78].

In another study, the 16-week administration of Formononetinin rats showed a potential ability to control nephropathy in type 2 diabetes [79]. Formononetin was also found to express antiosteosarcoma actions in both *in vitro* and *in vitro* studies [80].

Knowledge based on previous studies suggests that in long bones, bone loss is often observed in the proximal and distal positions and the change in the diaphysis is imperceptible [81]. A recent investigation of femoral bone changes in rats after OVX confirmed that there is an increased femoral periosteal apposition at one and two months post OVX, which appears helpful in supporting the growing body weight under the condition of induced osteoporosis [82]. The classical opinion regarding bone loss due to osteoporosis is questioned and suggests that OVX rats are capable of adapting to OVX-related alterations of bone properties [82]. According to the literature the effects of OVX on BMD and microarchitecture are rat age- and site- dependent [62, 83], which renders the field of diaphyseal femoral strength quite explorable. Bone-turnover is a procedure with long duration, therefore longer studies and multiple measurement points are needed [84] to further evaluate the above-mentioned correlations between OVX, BMD and bone strength.

It may be considered that our study has the following limitations: We intentionally did not include an additional group of Estradiol in accordance with the principle of Reduction of the the “3Rs” regarding the use of animals in scientific procedures [85]. Estradiol is widely studied and well known for its positive effect on BMD but also for its side effects on animals and humans. Our main goal was to investigate alternative to estradiol treatments focused on bone loss prevention. Furthermore due to limitations of our technical equipment, it was not possible to examine mechanical strength of the lumbar vertebrae.

## 5. Conclusion

In conclusion *Glycyrrhiza glabra* root extract notably protected tibial BMD loss in OVX + G rats in comparison to OVX rats, but did not improve biomechanical strength in femoral diaphysis, which was the only site tested for mechanical strength. Other bone sites need to be evaluated in future studies with this promising extract.

Conflicts of interest (applies to all authors): There are no relevant financial interests related to the material in the manuscript.
